# Mr. Ring, on Inoculation

**Published:** 1805-12

**Authors:** John Ring


### To the Editors of the Medical and Physical Journal.

GENTLEMEN,

DR. MOSELEY, in his Treatise on the Cow-pox, which he disingenuously confounds with the murrain, tells us, he has inoculated thousands in the West Indies, and in Europe, without losing a single patient. This is a question which I am not competent to decide; never having heard the name of any person inoculated by him: but having been told that a child of the Earl of Rosslyn had died of inoculation, under Dr. Moseley's care, I wrote to his lordship, who did me the honour to inform me, that he has had the misfortune to lose two children by the inoculated small-pox; that one of them was attended by Mr. Dundas, and, his lordship believed, by Dr. Moseley. The other was attended by Mr. Knight.

In order to investigate still further the particulars of the first case, I wrote to Mr. Dundas; who favoured me with the following particulars. In September 1792, Mr. Dundas was applied to by Mrs. Bouverie, the mother of the Countess of Rosslyn, to inoculate a son of her ladyship, six months old. He was troubled with a wheezing; on which account Mr. Dundas advised that he should not be inoculated. Dr. Moseley was then consulted; and having given his opinion that there was no reason why the operation should be postponed, Mr. Dundas had no longer any option. He was therefore inoculated; and had the confluent small-pox, of which he died on the eleventh day. Dr. Moseley was not present at the time of the inoculation; but saw him once or twice during the progress of the disease.

Mr. Dundas does not know, whether Dr. Moseley ever saw the child whom Mr. Knight inoculated; but every rational man will coincide in opinion with him, that when Dr. Moseley wrote on the subject of vaccination, with these cases fresh in his memory, it was natural to expect, he would have been rather less violent in his opposition to the practice.

A few days since, I received a letter on this subject from an eminent physician, in which he observes, that although a complete refutation of Dr. Moseley's pamphlet is published, "every person will not read it; and death and destruction are extending on every side. *In London, one child is killed by the small-pox every three hours, reck-*

*oning*

*oning day and night; and these murders go unpunished.*" My correspondent also expresses his "fear, that *the apostles of destruction preach harder than those of salvation.*"

One medical man, in the neighbourood of the metropolis, had a few days ago, two patients lying dead, slain by his lancet; and another was expected to share the same fate.

The following intelligence, which I lately received from a correspondent at Edinburgh, will, I doubt not, afford great satisfaction to many of your readers. "Vaccination, as far as I can judge, is here *universally adopted* A case of confluent small-pox occurred in the hospital few weeks since. Dr. Rutherford, the Clinical Professor, advised us, rather to regard it as a *curiosity* than as a case of importance; *as we should probably not see another.*

"Dr. Gregory, and all the other physicians and surgeons, whom I have heard deliver their opinions on the subject, speak of the annihilation of the small-pox with equal confidence. Dr. Gregory in particular, when lecturing on the small-pox, observed, that he should treat the subject very lightly, as he expected, in a few years, it would cease to exist in this country."

The following case of casual cow-pock is probably more ancient than any one on record, except that of the Duchess of Cleveland in the time of Charles II. It was communicated to me by Mrs. Richardson, housekeeper to Mr. Jervoise Clarke Jervoise. Her sister-in-law, daughter of the late Mr. Perry, pewterer, in Fore Street, informed Mrs. Richardson, that her mother had a disorder in her infancy, which a medical man assured her would prevent her from ever having the small-pox. Mrs. Perry died upwards of forty years ago, and was then about fifty years of age.

To this anecdote I shall add another, tending to shew how generally the opinion of the antivariolous property of the cow-pock has prevailed. Mrs. Reynolds, at No. 10, Stewart's Rents, Drury Lane, informed me, that two of her sisters, and a brother, had the casual cow-pox, twenty-seven years ago, at Newcastle, near Limerick. She often heard her relations say, that the cow-pox prevented the small-pox. Her sisters have frequently been exposed to the small-pox, since that time; each of them having had children labouring under that disease, one of whom died of it. Her brother was also exposed to the small-pox, twenty years after he had the cow-pock; but escaped infection.

I now

I now beg leave to add some other cases of the small-pox occurring a second time. Mrs. Norris, No. 4, South Street, Grosvenor Square, was inoculated for the small-pox at the age of five months; in consequence of which her arm rose, and she had an eruption of about fifteen pustules in different parts of the body. The cicatrix on the arm, and the marks of some of the surrounding pustules, are still visible.

Ten years afterwards, her sister had the small-pox in a severe manner, and was blind several days. Mrs. Norris caught the disease from her by contact, and had much previous indisposition; the eruptive fever being far more severe and alarming than that of her sister, although the eruption was less. One remarkable pit, the effect of this second eruption, is very conspicuous on her cheek. The medical practitioner who attended her, wished to take matter from her for the purpose of inoculation, but this she refused.

The foregoing case was communicated to me by Mr. Merriman, who very judiciously remarks, that it is one proof, in addition to many others, of local small-pox, attended with violent fever and general indisposition; in the same manner as those symptoms sometimes occur in persons who have undergone vaccination.

Miss Browne, daughter of Mr. Browne, in George Street, Adelphi, was inoculated for the small-pox at Exeter, by Mr. Cornish, together with three children of the Rev. Dr. Halloran, of Alphington. Her arm rose well, she sickened, and had an eruption; and was much more indisposed than either of the children inoculated with her; being very delirious about the turn of the disorder. Four years after, when residing in Bartlet's Buildings, she had the small-pox severely in the natural way, and was attended by the late Mr. Darley. She has many marks of this second attack remaining; and on this occasion, the children of the Rev. Mr. Wood, Chaplain to the Lord Mayor, were inoculated from her. The children of Dr. Halloran have hitherto resisted the infection of the small-pox.

Another case of the small-pox after inoculation occurred in a daughter of Mr. F. Albert, Page to the Queen. She was inoculated by that eminent surgeon, Mr. Bromfield, who not only paid particular attention to the practice, but also published a treatise on the subject, which is now in my possession. Mr. Bromfield was satisfied with the appearances, and pronounced her safe; but she afterwards wards caught the small-pox from her brother, and had a heavy burden.

I have now before me the copy of a letter from Dowager Lady Peyton to Dr. Jenner, dated Darsham House, July the 10th, 1805, in which her ladyship gives an account of her successful practice in vaccination; and subjoins the three following cases of persons who have had, or at least apparently had, the small-pox twice.

1. In the year 1777, a postillion, who lived in her ladyship's family, was not inoculated when the children and some of the servants underwent that operation, because he and his parents declared he had already been inoculated by Mr Framingham of Swaffham, and had an eruption of small-pox, attended with constitutional indisposition. Soon after the children and servants recovered, this young man was attacked with the small-pox, and had it very full.

2. In the year 1790 or 1791, a butler in the service of her ladyship's brother, who had been several years before inoculated by Mr. Mudd, a surgeon near Bury, was seized with the small-pox soon after his return from London, and had it in so violent a manner, that he narrowly escaped with his life.

3. Major Dode, formerly of the Suffolk Militia, who married a relation of her ladyship, was declared by his family to have had the natural small-pox twice; and both times severely.

Some little estimate of the frequency of failures in variolous inoculation, and of a recurrence of the small-pox, may be formed from the foregoing communication; when we consider that one individual, not of the medical profession, here furnishes three instances of the kind.

I have also had the pleasure of perusing a letter from Dr. Barboza to Dr. Jenner, dated Bay of All Saints, Jan. the 26th, 1805, in which he states, that having carried vaccine matter from England to Lisbon, and succeeded in exciting the disease, he furnished himself with a fresh supply when he sailed for Brazil, but it there failed to produce the desired effect. He therefore acted, as every one in such a situation ever will act, who has the least regard for the interests of humanity; he sent some boys to Lisbon, who being vaccinated in succession, conveyed the recent matter to Brazil.

Upwards of seven hundred people had been vaccinated since the 30th of December, in the Palace of the Governor Mr. Menezes, who, with a zeal that reflects on him great honour, had done every thing in his power to promote this this beneficial practice. He then intended to send matter to Rio Janeiro and Angola; and the Viscount of Anadia, Minister of the Marine, and of the Colonies, had given orders for that purpose to all the Governors of Brazil. Dr. Barboze is appointed Superintendant of Vaccination.

While attempts are daily made here at home, to crush the practice, by the grossest misrepresentations, and the most base and infamous falsehoods, it cannot be superfluous to add one other document, in order to prove, if any further proof is necessary, that in this instance, as well as others, the old adage is verified, 'that no prophet is honoured in his country.' It is part of a letter from Mrs. Clifton of Demarary, who some time ago came to England, on account of the education of her children; and, when at Cheltenham, was instructed in vaccination by Dr. Jenner. She also paid great attention to the practice while in London; and, on her return, carried matter back to that settlement. I had long ago transmitted some, which proved successful, as was already stated in your Journal; but Mrs. Clifton informed me, that after the garrisons of Demarary and Essequibo had been vaccinated, it was lost, the natives in general, who are of Dutch extraction, being prejudiced against inoculation.

On Mrs. Clifton's arrival at Demarary, she found all the faculty busy in inoculating for the cow-pock; and her own people had already undergone the operation. She observes that the patients under vaccination, in that climate, are more indisposed than in England.

I am, &c.

JOHN RING.

